# Free-Standing SnO_2_@rGO Anode via the Anti-solvent-assisted Precipitation for Superior Lithium Storage Performance

**DOI:** 10.3389/fchem.2019.00878

**Published:** 2019-12-19

**Authors:** Shuli Jiang, Ruiming Huang, Wenchang Zhu, Xiangyi Li, Yue Zhao, Zhixiang Gao, Lijun Gao, Jianqing Zhao

**Affiliations:** ^1^College of Energy, Soochow Institute for Energy and Materials Innovations, Soochow University, Suzhou, China; ^2^Key Laboratory of Advanced Carbon Materials and Wearable Energy Technologies of Jiangsu Province, Soochow University, Suzhou, China; ^3^Department of Chemistry, Rutgers-Newark, The State University of New Jersey, Newark, NJ, United States

**Keywords:** SnO_2_, rGO, anti-solvent-assisted precipitation, free-standing anode, lithium-ion battery

## Abstract

Metal oxides have been attractive as high-capacity anode materials for lithium-ion batteries. However, oxide anodes encounter drastic volumetric changes during lithium ion storage through the conversion reaction and alloying/dealloying processes, leading to rapid capacity decay and poor cycling stability. Here, we report a free-standing SnO_2_@reduced graphene oxide (SnO_2_@rGO) composite anode, in which SnO_2_ nanoparticles are tightly wrapped within wrinkled rGO sheets. The SnO_2_@rGO sheet is assembled in high porosity via an anti-solvent-assisted precipitation of dispersed SnO_2_ nanoparticles and graphene oxide sheets in the distilled water, followed by the filtration and post-annealing processes. Significantly enhanced lithium storage performance has been obtained of the SnO_2_@rGO anode compared with the bare SnO_2_ anode material. A high charge capacity above 700 mAh g^−1^ can be achieved with a satisfying 95.6% retention after 50 cycles at a current density of 500 mA g^−1^, superior to reserved 126 mAh g^−1^ and a much lower 16.8% retention of the bare SnO_2_ anode. XRD pattern and HRTEM images of the cycled SnO_2_@rGO anode material verify the expected oxidation of Sn to SnO_2_ at the fully-charged state in the 50th cycle. In addition, FESEM and TEM images reveal the well-preserved free-standing structure after cycling, which accounts for high reversible capacity and excellent cycling stability of such a SnO_2_@rGO anode. This work provides a promising SnO_2_-based anode for high-capacity lithium-ion batteries, together with an effective fabrication adoptable to prepare different free-standing composite materials for device applications.

## Introduction

With the rapid development of portable electronic devices, pure electric vehicles and emerging large-scale energy storage systems, lithium-ion batteries are required to at least have high energy and power densities, in order to meet high-grade demands for various practical applications. The exploration of alternative anode materials has become an urgent task to pursue high lithium storage capacity, together with excellent rate capability and cycling stability, because the specific capacity of the commercial graphite anode has been reached to the theoretical limit of 372 mAh g^−1^. The tin-based oxides have been widely reported as promising anode candidates, due to the high capacity, non-toxicity, and natural abundance (Hu et al., 2017; Sahoo and Ramaprabhu, 2018; Cao et al., 2019; Hong et al., 2019). As reported in the literature (Zhao et al., 2016; Ahmed et al., 2017; Cui et al., 2017), lithium storage capacity of the SnO_2_ anode material is on the basis of reversible alloying/dealloying processes of Sn_x_Li (0 < *x* ≤ 4.4, corresponding to the maximum theoretical capacity of 782 mAh g^−1^ when the *x* = 4.4) after an initial irreversible conversion reaction from original SnO_2_ to the metallic Sn (Wang et al., 2012). An impressive capacity up to 1,493 mAh g^−1^ based on *x* = 8.4 has been achieved by Wang and co-authors (Wang et al., 2015) through realizing the fully reversible oxidation from the reduced Sn back to SnO intermediate then to SnO_2_. In addition, increased capacities can also be obtained via a partial Sn to SnO_2_ conversion, coupled with synergistic effects from different carbonaceous materials or functional nanomaterials (Kim et al., 2014, 2017; Sun et al., 2015). However, the practical application of SnO_2_-based anode materials is impeded by severe volumetric expansion/contraction up to 259% during alloying/dealloying processes of Sn_x_Li variants, leading to the structural degradation and poor electronic conductivity of the anode. Additionally, the undesirable aggregation of reduced Sn nanoparticles into clusters together with the Sn pulverization occurs during prolonged electrochemical cycling, which further brings about the deactivation of active Sn particles, and thus the rapid capacity loss and poor cycling stability (Li et al., 2015; Liu et al., 2015; Min et al., 2019).

Compared with extensively-reported strategies, such as reducing particles size (Park et al., 2007; Wang et al., 2015; Xia et al., 2016; Yao et al., 2019) and dispersing active anode materials into a solid matrix (Xu et al., 2012; Zheng et al., 2016), few attention has been devoted to the binders in the anode (Zhang et al., 2014). The traditional binder, i.e., polyvinylidene fluoride (PVDF) is insulating and electrochemically inactive, which is used to strengthen mechanical connections between active anode materials, conductive additives, and the current collector. However, the presence of the binder decreases the overall electronic conductivity, but increases the electrochemical polarization in the anode. As reported in the literature (Kumar et al., 2019; Pan et al., 2019), polymetric conductive binders with strong mechanical binding force and even self-healing capability have been demonstrated to address detrimental volume effects of oxide/metal-based anode materials. Moreover, such polymetric binders play an additional role in offering the desired pathway for the charge transfer, resulting in free conductive carbon additives in the anode. However, the synthesis of those conductive polymers is expensive and time-consuming, which requires complex coupling reactions, noble metal catalysts and stringent reaction conditions. In addition to the binders, the metallic current collector, i.e., the copper foil is also needed for the anode fabrication. Within the typical anode, inactive components involving the binder, conductive additive and current collector exceed 50 wt.% of the total electrode mass. Therefore, an advanced electrode structure should be rationally designed to increase the energy density of the anode.

The graphene and reduced graphene oxide (rGO) have been extensively used for energy storage and conversion applications, especially in lithium-ion batteries, owing to their unique two-dimensional structures with excellent flexibility, mechanical strength, chemical stability, and thermal and electronic conductivities (Rong et al., 2014; Deng et al., 2016; Ahn et al., 2019; Riyanto et al., 2019). Both graphene and rGO materials have been demonstrated to act as reliable supporting and buffering matrixes to improve electrochemical performance of high-capacity anode materials, such as SnO_2_ and Si, by accommodating their drastic volume changes during the lithium storage (Jiang et al., 2017; Ma et al., 2017; Chen et al., 2018; Deng et al., 2019). Tri-dimensional hybrid materials consisting of graphene (or rGO) sheets and active anode particles can be served as promising free-standing anodes with free conductive additives and binders. The wrinkled structure of graphene or rGO sheets may be also fabricated, in order to ensure the “buffering” capability. On the other hand, active particles are required to distribute in the graphene-based matrix uniformly (Li et al., 2015; Wang et al., 2018). As reported in the literature (Li et al., 2011, 2019), either aerosol spray drying process or solution ionic strength engineering has been demonstrated as an effective route to obtain desired composite materials, but the conductive additive and polymer binder are still added for the electrode preparation. It would be very interesting to explore assembly methods for the preparation of free-standing graphene-based anodes full of pores and wrinkles, in which active particles are uniformly distributed free of the conductive carbon and binder components, resulting in the maximum capacity contribution of such the anode (Xia et al., 2019; Xing et al., 2020).

In this work, we report an effective approach to fabricate a free-standing SnO_2_@rGO composite anode through an anti-solvent-assisted precipitation followed by the suction filtration. The resulting SnO_2_@rGO anode has sufficient wrinkles and internal channels, which are expected to favor not only the electrolyte permeation but also the accommodation of large volume expansion during cyclic lithium storage of the SnO_2_ anode material, and thus contribute to enhanced electrochemical performance compared with the bare SnO_2_ anode material. The assembly method developed in this study may be adopted to prepare different free-standing composite materials consisting of a flexible matrix and functional nanoparticles for device applications beyond lithium-ion batteries.

## Experimental

### Preparation of the Graphene Oxide (GO) and Free-Standing SnO_2_@rGO Electrode

Graphene oxide was prepared via a modified Hammond method (Marcano et al., 2010). Typically, 0.75 g graphite flakes and 4.5 g KMnO_4_ were added to a 100 mL concentrated H_2_SO_4_ and H_3_PO_4_ solution at a volume ratio of 90:10. After heating at 50°C for 12 h under continuous stirring, the mixed solution was cooled to room temperature and poured onto a 100 mL ice with 1 mL 30 wt.% H_2_O_2_. The obtained slurry was then centrifuged and washed repeatedly with 10% HCl to remove Mn^2+^ ion, followed by removing the majority of Cl^−^ ion via the successive washing using the acetone. For the complete removal of all the ions, deionized water was used to wash GO until no precipitate was observed when the GO solution was mixed the 10 mM AgNO_3_ solution.

In order to prepare the free-standing SnO_2_@rGO electrode, 4 mg SnO_2_ (Sigma Aldrich) and 8.8 mg GO were co-dispersed in 15 mL de-ionized water for 15 min. Twenty milligrams LiCl powder was directly added. The suspension was then sonicated for an additional 1 min. Sixty milliliters acetone was poured into the above suspension at one time shot. The resulted mixture was then collected via vacuum filtration using the PTFE filter paper with the pore size of 0.2 μm and the diameter of 15 mm (Sterlitech). The obtained free-standing SnO_2_@GO sheet on the PTFE paper was then together dried in vacuum at 60°C for 2 h. After peeling off from the PTFE paper, the dried SnO_2_@GO sheet was subjected to post-annealing processes to reduce the GO and obtain SnO_2_@rGO. The heating temperature was increased 100°C per step with a duration time of 1 h up to 400°C, followed by heating at 500°C for 3 h in the Ar flow. The typical mass of as-prepared SnO_2_@rGO electrode is 5.6 mg, corresponding to the loading density of 3.1 mg/cm^2^

### Material Characterizations

Crystallographic structures of as-prepared materials were identified by X-ray diffraction (XRD) on a Bruker D8 Advance automatic diffractometer with Cu Kα radiation. Morphology and structure of different samples were observed by using scanning electron microscopy (SEM, Hitachi S-4800) and transmission electron microscopy (TEM, FEI Tecnai G2T20) at an acceleration voltage of 200 kV, respectively. The chemical environment and valent states of anions and cations within different materials were characterized by X-ray photoelectron spectroscopic (XPS) measurements on an ESCALAB 250Xi XPS equipment. All XPS spectra were calibrated according to the binding energy of the C 1s peak at 284.8 eV. The degree of graphitization of the GO and rGO materials were characterized by Raman spectra on a Horiba JY LabRAM Aramis equipment. The rGO content within the SnO_2_@rGO electrode was determined by the thermogravimetric analysis on a TG/DTA-7300 thermal analyzer (Seko) in air flow at a temperature range between room temperature and 900°C.

### Electrochemical Measurements

Electrochemical measurements were carried out in a two-electrode system for lithium-ion battery testing. The free-standing SnO_2_@rGO sheet was directly used as the working anode. All CR2025-type coin cells were assembled in an Ar-filled glove box, using the lithium metal as the counter and reference electrode, and Celgard3501 as the separator. The electrolyte was 1 M LiPF_6_ dissolved in a mixture of ethylene carbonate (EC), dimethyl carbonate (DMC) and diethyl carbonate (DEC) at a volumetric ratio of 4:3:3. Galvanostatic charge/discharge of the cells were performed on the MTI BST8-MA-battery analyzer in a voltage range of 0.01–3.0 V vs. Li^+^/Li. Cyclic voltammetric (CV) curves were recorded at a scanning rate of 0.1 mV s^−1^ between 0.01 and 3 V vs. Li^+^/Li, and electrochemical impedance spectroscopy (EIS) was conducted from the open circuit voltage of testing cells in a frequency range of 10 mHZ-100 kHZ with an AC amplitude of 5 mV using an electrochemical analyzer (CHI 760C).

## Results and Discussion

The SnO_2_ oxide has been extensively reported as a high-capacity anode material for lithium-ion batteries, but suffers from fast capacity decay during cycling, due to drastic volume changes for the lithium storage on the basis of the reversible initial conversion reaction (Equation 1) and successive alloying/dealloying processes (Equation 2) as follows (Huang et al., 2010; Wang et al., 2011; Zhang et al., 2012; Jiang et al., 2017):

(1) $$\begin{array}{lll} 4Li^{+} & + & SnO_{2}+4e^{-}↔2Li_{2}O+Sn\;\left(\text{capacity delivered}:\text{711 mAh}/\text{g}\right) \end{array}$$

(2) $$\begin{array}{lll} xLi^{+} & + & Sn+xe^{-}↔Li_{x}Sn\;\left(0\leq x\leq 4.4\right)\;\left(\text{capacity delivered}:\text{782 mAh}/\text{g}\right) \end{array}$$

**Figure 1:**
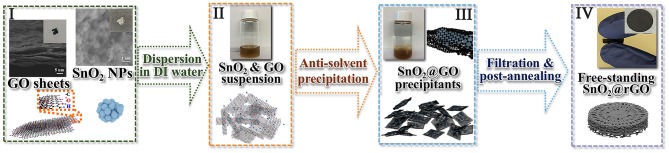
Schematic illustrations for the synthesis of the free-standing SnO_2_@rGO sheet.

However, it is difficult to maintain the full reversibility of the conversion reaction as shown in Equation (1), resulting in a capacity range of 782–1,493 mAh/g for the SnO_2_ anode material (Wang et al., 2013; Deng et al., 2016; Cao et al., 2019). In order to obtain the maximum capacity with excellent cycling stability, a carbon-based framework should be employed to support active SnO_2_ nanoparticles by serving as a conductive network and a structural cushion to release mechanical strains during lithiation/delithiation of such the oxide anode. In addition, the uniform dispersion of SnO_2_ nanoparticles in the carbonaceous framework is also highly required to restrict from the unfavorable aggregation and pulverization of the reduced Sn nanoparticles. The as-prepared GO nanosheets are here used to accommodate commercial SnO_2_ nanoparticles, and a free-standing SnO_2_@rGO anode is obtained through an effective anti-solvent-assisted precipitation followed by a post-annealing process. Figure 1 shows schematic illustrations for the synthesis of the free-standing SnO_2_@rGO sheet, together with photographs taken in different stages. Notably, an anti-solvent-assisted precipitation, which is inspired by a frequently used method to purify and/or concentrate RNA and DNA in biochemistry (Zeugin and Hartley, 1985), is adopted to assemble the SnO_2_@GO composite material. The synergy of ionic strength and dielectric constant change induced by a low concentrated salt and an organic solvent (such as ethanol and acetone) allows for the controllable assembly of well-dispersed graphene oxide (GO) nanosheets and SnO_2_ nanoparticles in an aqueous solution. SEM images as shown at **Stage I** reveal distinct aggregations of both original GO nanosheets and SnO_2_ nanoparticles. The brown solution-like suspension can be obtained through simply dispersing GO and SnO_2_ powders in the deionized (DI) water by the ultrasonic treatment at **Stage II**, on account of numerous hydrophilic groups involving -COOH and -OH ends at the surface of GO sheets and nano-sized SnO_2_ particles. The assembly of GO sheets and SnO_2_ nanoparticles can be implemented at **Stage III** by adding a small amount of LiCl salt and subsequently the acetone as an anti-solvent, resulting in the rapid precipitation as shown in the vial. The instant formation of SnO_2_@GO precipitant is attributed to the considerably strengthened electrostatic force between negatively-charged GO sheets and positively-charged Li^+^ ion induced by the anti-solvent acetone. Thus, the stable GO dispersion is disturbed, and GO sheets crumple and fold to minimize the surface energy. With the co-existence of well-distributed SnO_2_ nanoparticles in the GO solution, SnO_2_ nanoparticles are all wrapped in wrinkled GO nanosheets, resulting in the SnO_2_@GO precipitation. As shown at **Stage IV**, the free-standing SnO_2_@rGO sheet is obtained via the facile vacuum filtration of all SnO_2_@GO precipitants, followed by a post-annealing process.

**Figure 2:**
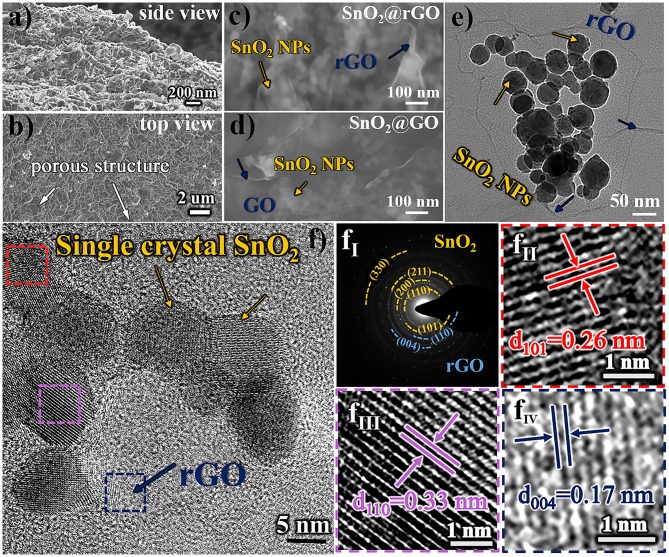
Morphologic and structural characteristics of resulting SnO_2_@rGO composite material: SEM images of **(a)** the side view and **(b)** the top view, **(c)** zoom-in FESEM image in comparison with **(d)** the original SnO_2_@GO material before the reduction, **(e)** TEM image, **(f)** HRTEM image, coupled with **(f**_**I**_**)** SAED pattern and **(f**_**II**_**–f**_**IV**_**)** enlarged lattice fringes as squared in red, pink and navy dashed lines, respectively.

**Figure 3:**
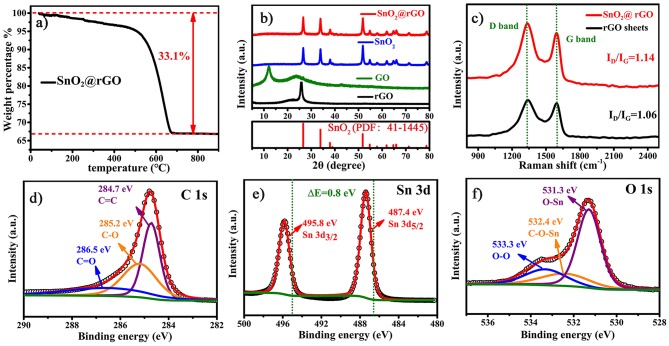
Composition, crystallographic structure, and chemical environment of the SnO_2_@rGO composite material: **(a)** TG analysis to determine the content of SnO_2_ component, **(b)** XRD patterns, **(c)** Raman spectra compared with rGO sheets, and characteristic XPS spectra of **(d)** C 1s, **(e)** Sn 3d, and **(f)** O 1s.

Morphologic and structural characteristics of resulting SnO_2_@rGO composite material are examined in Figure 2. Figures 2a,b show SEM images taken at the intersection area (the side view) and the surface (the top view) of the free-standing SnO_2_@rGO sheet, respectively, indicating a distinct porous structure. The zoom-in FESEM image as shown in Figure 2c clarifies that all SnO_2_ nanoparticles are tightly wrapped within rGO sheets full of expected wrinkles, which is very similar to the assembled structure of original SnO_2_@GO material (Figure 2d). The post-annealing process only results in the reduction of GO component to rGO material, while encased SnO_2_ nanoparticles are stabilized without the particle growth and phase reduction to either SnO or Sn (will be discussed in Figure 3). As captured in TEM observation (Figure 2e), SnO_2_ nanoparticles all show spherical shapes in a particle size range of 10–50 nm. The HRTEM image of the SnO_2_@rGO material is also captured as displayed in Figure 2f, coupled with corresponding selected area electron diffraction (SAED) pattern as shown in Figure 2f_I_, indicating the single crystal property of SnO_2_ particles and a high graphitization degree of the rGO sheet. Lattice fringes of two selected SnO_2_ particles (squared in red and pink dashed lines in Figure 2f) with the corresponding *d*-space distance of 0.26 and 0.33 nm can be indexed to (101) and (110) planes of the SnO_2_ in a tetragonal rutile structure, as shown in Figures 2f_II_, f_III_, respectively. Additionally, Figure 2f_IV_ shows the enlarged selected lattice fringe of the rGO sheet (squared in the navy dashed line in Figure 2f), corresponding to (004) planes of the hexagonal graphite phase with an interplanar distance of 0.17 nm. Overall, SEM and TEM observations demonstrate the desired structural integrity of the free-standing SnO_2_@rGO sheet with the high porosity.

Figure 3 further analyzes the composition, crystallographic structure and chemical environment of the SnO_2_@rGO composite material. The rGO content within the SnO_2_@rGO is determined to be 33.1 wt.%, according to the TG analysis as shown in Figure 3a. However, XRD pattern of the SnO_2_@rGO is identical to that of the pure SnO_2_ powder (Figure 3b), which can be well indexed to the rutile SnO_2_ phase in the tetragonal structure (PDF: 41–1445). The absence of characteristic diffraction peaks from the rGO component implies the homogeneous dispersion of SnO_2_ nanoparticles between each rGO layer, in accordance with SEM and TEM observations (Figures 2c,e). By contrast, the typical (002) peak can be detected at 2θ = 12^0^ in the XRD pattern of GO powder, indicating the ordered stack of original GO sheets. The other broad peak located at 2θ = 23^0^ is resulted from the partial reduction of the GO material, possibly owing to the drying process during the material collection. Accordingly, the Raman spectrum of rGO sheets shows an intensity ratio of the D band over G band, i.e., the I_D_/I_G_ equal to 1.06, also indicating a certain degree of graphitization, because the G band peak located near 1,589 cm^−1^ is related to the vibration of *sp*^2^-bonded carbon atoms in an ordered two-dimensional hexagonal lattice of carbon-based materials, while the D band peak around 1,339 cm^−1^ is associated with defects and disorder formed in the hexagonal graphitic layers (Wang et al., 2012). However, it is interesting to see that the rGO component in the SnO_2_@rGO composite material gives a higher ratio of I_D_/I_G_ = 1.14, although it was subjected to the post-annealing process for reducing defects in original GO sheets. The increased disordered domains in the rGO component are probably caused by wrapped SnO_2_ nanoparticles through the possible chemical bonding; hence, XPS spectra were carried out on the SnO_2_@rGO powder to study chemical environments of Sn, O, and C elements. Characteristic C 1s, Sn 3d, and O 1s XPS peaks are plotted in Figures 3d–f, respectively. According to fitting patterns, the C 1s peak can be deconvoluted to one dominant C=C contribution at 284.7 eV, together with two weak effects from the C-O at 285.2 eV and C=O at 286.5 eV (Figure 3d), indicating a typical chemical environment of the rGO component as reported in the literature (Min et al., 2019). It is worth noting that two Sn 3d XPS peaks in Figure 3e shift to the higher binding energies for 0.8 eV compared with the reported pure SnO_2_ powder (Yao et al., 2019). Furthermore, different contributions of O-Sn bond at 531.3 eV, Sn-O-C bond at 532.4 eV and O-O bond at 533.3 eV can be identified in the O 1s peak on the basis of fitting patterns as shown in Figure 3f, respectively. Thus, Raman and XPS spectra together indicate the favorable chemical bonding formed between SnO_2_ and rGO components within the SnO_2_@rGO composite material.

**Figure 4:**
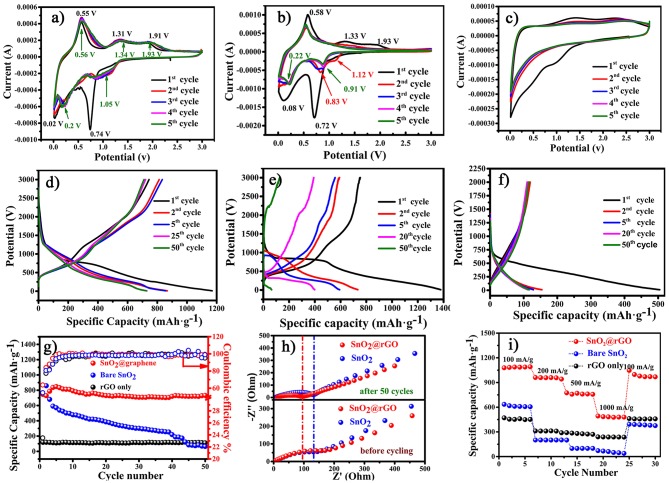
Electrochemical performance of the free-standing SnO_2_@rGO anode in a voltage range of 0.01–3.0 V vs. Li^+^/Li: CV curves of **(a)** SnO_2_@rGO, **(b)** bare SnO_2_, and **(c)** bare rGO anodes in the first five cycles at a scanning rate of 0.1 mV s^−1^, galvanostatic charge/discharge curves in different cycles at a current density of 500 mA g^−1^ of **(d)** SnO_2_@rGO, **(e)** bare SnO_2_, and **(f)** bare rGO anodes, **(g)** cycling performance at a current density of 500 mA g^−1^, **(h)** EIS spectra of both the bare SnO_2_ and SnO_2_@rGO anodes before and after cycling and **(i)** comparative high-rate performance of three anodes.

The resulting SnO_2_@rGO sheet (Figure 1IV) is directly used as the free-standing anode for lithium storage performance evaluations without adding the conductive carbon, polymer binder and even the copper current collector. For the comparative propose, the bare rGO film is also fabricated as the other free-standing anode, and the bare SnO_2_-based anode is composed of 70 wt.% SnO_2_ nanoparticles as the active material, 20 wt.% acetylene black as the conductive carbon and 10 wt.% polyvinylidene fluoride (PVDF) as the binder. Figures 4a–c show cyclic voltammetric (CV) curves of the SnO_2_@rGO, SnO_2_ and bare rGO anodes in the first five cycles recorded at a scanning rate of 0.1 mV s^−1^ in a voltage range of 0.01–3.0 V vs. Li^+^/Li. As referred to CV characteristics of bare rGO and SnO_2_ anode materials, the initial cathodic peak at 0.74 V in the first CV discharge of the SnO_2_@rGO anode is attributed to the conversion reaction of SnO_2_ to the metallic Sn and Li_2_O, as described in Equation (1). The subsequent broad cathodic peak below 0.5 V reveals combined effects from the successive formation of Li_x_Sn (0 < *x* < 4.4) alloys (Equation 2), lithiation of the rGO material (Figure 4b) and the formation of solid electrolyte interphase (SEI) film at the surface of the working electrode. Correspondingly, a weak anodic shoulder peak at 0.2 V in the following charge process can be probably assigned to the reversible delithiation from the rGO component. The dominant anodic peak positioned at 0.55 V corresponds to the dealloying process of as-formed Li_x_Sn compounds. It is interesting to find a very wide anodic park cross a voltage range between 1.0 and 2.5 V. As reported in the literature (Chen and Yano, 2013; Kim et al., 2014; Hong et al., 2019), such electrochemical CV behaviors result from the reversible reaction from the reduced Sn to the SnO intermediate near 1.3 V and then to the SnO_2_ near 1.9 V. Accordingly, a wide anodic peak appears in a voltage range of 0.75–1.5 V in the second CV cycle, which supports our speculation of the reversible conversion reaction between SnO_2_ and Sn as shown in Equation (1). Two following cathodic peaks located at 0.2 and 0.01 V are ascribed to the alloying process of Li_x_Sn and lithium ion storage of the rGO, respectively, together with the continuous growth of SEI film. Notably, CV profiles of this SnO_2_@rGO composite anode in next cycles are almost identical to each other after initial electrochemical activations in the first two cycles, indicating the excellent electrochemical reversibility similar to that of the bare rGO anode (Figure 4c). By contrast, the reversible SnO_2_ ↔ SnO ↔ Sn conversion reaction cannot be well-maintained in the bare SnO_2_ anode, leading to the gradual disappearance of anodic/cathodic CV peaks around 1.5/1.0 V in its CV curves after initial two cycles (Figure 4b), which may account for the drastic capacity decay of the bare SnO_2_ anode material in initial 10 of charge/discharge cycles. In a short summary, CV results indicate the satisfied electrochemical reversibility for stable lithium ion storage of the free-standing SnO_2_@rGO anode on the basis of synergistic effects from SnO_2_ and rGO components (Cong et al., 2015; Huang et al., 2016).

Figures 4d,f plot galvanostatic charge/discharge curves at different cycles of these three anodes at a current density of 500 mA g^−1^ between 0.01 and 3.0 V vs. Li^+^/Li, and corresponding cycling performance are compared as shown in Figure 4g. The SnO_2_@rGO anode can deliver initial discharge and charge capacities of 1,169 and 744 mAh g^−1^ in the first cycle, respectively. The moderate initial columbic efficiency of 63.6% is mainly attributed to the undesirable SEI formation and the lithium consumption during the conversion reaction from SnO_2_ to metallic Sn and lithiated Li_2_O in the first discharge process. By contrast, the lower columbic efficiency of 54.6% in the bare SnO_2_ anode may result from the limited reversibility from the reduced Sn and Li_2_O back to SnO_2_ and Li^+^ ion in the charge process, and the inferior columbic efficiency of 23.5% in the bare rGO anode should be caused by considerably aggravated side reactions. As a result, the SnO_2_@rGO anode can retain a desired charge capacity of 711 mAh g^−1^ in the 50th cycle, corresponding to the capacity retention of 95.6%, much higher than that of the bare SnO_2_ anode (reserved 126 mAh g^−1^ and 16.8% retention in the 50th cycle as shown in Figure 4g). Significantly enhanced cycling stability of the SnO_2_@rGO anode can be attributed to the free-standing structure, in which active SnO_2_ nanoparticles are well-accommodated in the flexible rGO buffer with excellent conductivity and sufficient porosity. Figure 4h compares EIS spectra of both the bare SnO_2_ and SnO_2_@rGO anodes before and after cycling. The SnO_2_@rGO composite anode shows a distinctly lower charge-transfer resistance after 50 cycles compared with that of the bare SnO_2_ anode, which can be attributed to high electronic conductivity of the rGO framework even without any carbon additives. In addition, side reactions of active SnO_2_ and/or Li_x_Sn materials may also be impeded in the composite anode through the rGO protection. It is noting that the SnO_2_@rGO anode has the reduced resistance after cycling in comparison with its original state, possibly owing to the electrolyte infiltration into its internal structure during cycling. Furthermore, improved rate capability is also achieved of the SnO_2_@rGO anode, which delivers high charge capacities of 1,085, 958, 758, and 480 mAh g^−1^ at gradually-increased current densities of 100, 200, 500, and 1,000 mA g^−1^, respectively. The specific capacity around 1,000 mAh g^−1^ can be reversed, when such the free-standing anode is cycled back to a low current density of 100 mA g^−1^ after high-rate trials (Figure 4i). Electrochemical performance verifies that the free-standing SnO_2_@rGO anode supported by the rGO framework results in enhanced cycling stability and rate capability of SnO_2_-based anode materials for superior lithium storage.

**Figure 5:**
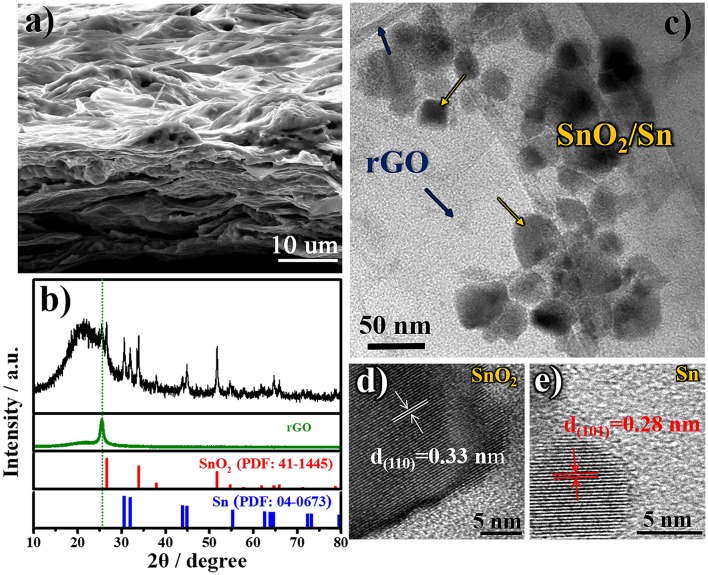
Morphologic and structural characteristics of cycled SnO_2_@rGO composite anode material in the fully-charged state after 50 cycles at a current density of 500 mA g^−1^: **(a)** FESEM image, **(b)** XRD pattern, **(c)** TEM image, and HRTEM images of **(d)** SnO_2_ and **(e)** Sn particles, respectively.

In order to deeply understand the relationship between the designed structure and superior electrochemical performance of free-standing SnO_2_@rGO composite material, the cycled anode is reexamined after 50 cycles in the full-charged state. Figure 5a shows the SEM image of cycled SnO_2_@rGO anode, which preserves the well free-standing structure. Tin-based nanoparticles are expectedly localized between rGO sheets as shown in the cross-section view. The cycled electrode shows compressed pores and channels by contrast with the initial structure (Figures 2a,b), which can be attributed to the SEI formation during cycling, together with the high-pressure effect from the coin cell assembly on the anode. As shown in Figure 5b, XRD pattern of the cycled anode reveals the co-existence of three rGO, SnO_2_ and Sn components in the fully-charged state. The reformation of SnO_2_ component verifies the partial oxidation of Sn to SnO_2_ during the charge reaction, in consistence with CV results (Figure 4a), which significantly accounts for high lithium storage capacity of the SnO_2_@rGO anode as illustrated in Equation (1). TEM and HRTEM images are recaptured on the cycled SnO_2_@rGO material. The TEM observation in Figure 5c clarifies the structural stability of such an assembled composite material, in which tin-based nanoparticles are uniformly and tightly wrapped within rGO sheets, in accordance with the SEM image (Figure 5a). Figures 5d,e presents HRTEM images of SnO_2_ and Sn particles through indexing their different lattice fringes, respectively, which is consistent with the XRD results (Figure 5b). All characterizations of the cycled anode demonstrate the desired structural stability and the Sn to SnO_2_ oxidation reaction during the charge reaction of the free-standing SnO_2_@rGO anode; hence, improved cycling stability with high specific capacity can be obtained for prolonged cycles.

## Conclusions

An anti-solvent precipitation method has been developed to effectively assemble SnO_2_ nanoparticles and graphene oxide sheets for the fabrication of a free-standing electrode, which is free of the conductive additive, polymer binder and current collector. The resulting SnO_2_@rGO composite anode shows significantly improved lithium storage performance compared with the bare SnO_2_ anode material. It can deliver an impressive charge capacity of ~500 mAh g^−1^ at a high current density of 1 A g^−1^, and an attractive capacity above 700 mAh g^−1^ can be retained after 50 cycles at a moderate current density of 500 mA/g. Enhanced cycling stability and rate capability of the composite anode can be attributed to the unique free-standing structure, in which all SnO_2_ nanoparticles are tightly wrapped within rGO sheets full of wrinkles. Material characterizations of the cycled anode indicate the desired structural stability of such a free-standing SnO_2_@rGO anode, accounting for superior lithium storage performance. This work offers a facile assembly method for the preparation of free-standing composite materials with enhanced performance for device applications.

## Data Availability Statement

The datasets generated for this study are available on request to the corresponding author.

## Author Contributions

JZ, LG, and RH conceived the idea and designed the work. SJ, RH, WZ, XL, YZ, and ZG carried out materials synthesis, characterizations, and performance measurements. SJ, JZ, and RH wrote and revised the paper. All authors have made substantial, direct and intellectual contributions to the work.

### Conflict of Interest

The authors declare that the research was conducted in the absence of any commercial or financial relationships that could be construed as a potential conflict of interest.
